# The effects of state rules on opioid prescribing in Indiana

**DOI:** 10.1186/s12913-018-2830-6

**Published:** 2018-01-18

**Authors:** Morhaf Al Achkar, Shaun Grannis, Debra Revere, Palmer MacKie, Meredith Howard, Sumedha Gupta

**Affiliations:** 1 University of Washington School of Medicine, Seattle, WA USA; 2 Indiana University School of Medicine, Indianapolis, IN USA; 3 Regenstrief Institute, Indianapolis, IN USA; 4 University of North Texas System College of Pharmacy, Fort Worth, TX USA; 5 Indiana University-Purdue University School of Liberal Art, Indianapolis, IN USA; 6 University of Washington School of Public Health, Seattle, WA USA

**Keywords:** Drug and opioid control, Drug monitoring, Drug overdose, Drug prescriptions, Health policy, Legislation, drug, Opioid-related disorders, Practice patterns, physicians’, Prescription drug misuse

## Abstract

**Background:**

Prescription opioids have been linked to over half of the 28,000 opioid overdose deaths in 2014. High rates of prescription opioid non-medical use have continued despite nearly all states implementing large-scale prescription drug monitoring programs (PDMP), which points to the need to examine the impact of state PDMP’s on curbing inappropriate opioid prescribing. In the short-term, PDMPs have been associated with short-term prescribing declines. Yet little is known about how such policies differentially impact patient subgroups or are interpreted by prescribing providers. Our objective was to compare volumes of prescribed opioids before and after Indiana implemented opioid prescribing emergency rules and stratify the changes in opioid prescribing by patient and provider subgroups.

**Methods:**

An interrupted time series analysis was conducted using data obtained from the Indiana PDMP. Prescription level data was merged with census data to characterize patient socioeconomic status. Analyses were stratified by patients’ gender, age, opioid dosage, and payer. The primary outcome indicator was the total morphine equivalent dose (MED) of dispensed opioids per day in the state of Indiana. Also considered were number of unique patients, unique providers, and prescriptions; MED per transaction and per day; and number of days supplied.

**Results:**

After controlling for time trends, we found that total MED for opioids decreased after implementing the new emergency rules, differing by patient gender, age, and payer. The effect was larger for males than females and almost 10 times larger for 0–20 year olds as compared to the 60+ age range. Medicare and Medicaid patients experienced more decline in prescribing than patients with private insurance. Patients with prescriptions paid for by workers’ comp experienced the most significant decline. The emergency rules were associated with decline in both the number of prescribers and the number of day supply.

**Conclusions:**

Although the Indiana opioid prescribing emergency rules impacted statewide prescribing behavior across all individual patient and provider characteristics, the emergency rules’ effect was not consistent across patient characteristics. Further studies are needed to assess how individual patient characteristics influence the interpretation and application of state policies on opioid prescribing.

## Background

Prescription opioids have been linked to over half of the 28,000 opioid overdose deaths in 2014, more than any year on record [1]. Every year, hundreds of millions of opioid prescriptions continue to be written, despite almost all states having large scale prescription drug monitoring programs (PDMP) and several states having “Must access” emergency rules in place [2]. At the end of 2013, Indiana implemented emergency rules to regulate opioid prescribing [3].

High rates of opioid prescribing in recent years has raised renewed interest in quantifying the success of state policies, in particular the latest emergency rules, in curbing inappropriate opioid prescribing. Early studies examining the impact of such policies reported significant initial decreases in opioid-related morbidity and mortality associated with decreased prescribing [4–6]. However, little is known about how the emergency rules differentially impact patient subgroups. Prior research has identified patient characteristics, such as gender, age, insurance type, and socioeconomic status as significant predictors of pain management, opioid prescribing, and drug non-medical use [7, 8]. In addition, research has documented issues attributed to biases in prescribing, such as: 1) undertreating pain in some patient subgroups, thus worsening an existing disparity in pain management [7, 8], and 2) failing to correct overprescribing for other patient groups, thus exposing them to the harmful effects of opioid use and non-medical use [9].

We sought to investigate the impact of Indiana’s new emergency prescribing rules on prescription of opioids. Our study aimed to: 1) compare volumes of prescribed opioids before and after the Indiana emergency rules; and 2) stratify the changes in opioid prescribing by patient and provider subgroups. Specifically, differential impacts of the emergency rules by patient gender, age, payer and zip code level aggregate measures of socioeconomic status are considered.

## Methods

### Data

Data were obtained from the Indiana Prescription Drug Monitoring Program (PDMP), which is called the Indiana’s Prescription Electronic Collection and Tracking Program (INSPECT) [10]. INSPECT records contain prescription-level, limited data set that include patient ID, patient zip code, gender, birth year, date the prescription was written, date the prescription was dispensed, quantity dispensed, number of days’ supplied, pharmacy ID, pharmacy zip code, provider ID, provider zip code, payer, National Drug Code (NDC), and drug name. All drug strengths were converted to the Morphine Equivalent Dose (MED) to facilitate comparison [11, 12].

### The Indiana emergency rules

Indiana implemented emergency prescribing rules that went into effect on December 15, 2013, and then became permanent in November 6, 2014 [3]. The rules are only applicable if patient has been prescribed, for more than three consecutive months: 1) >60 opioid-containing pills per month; or 2) A morphine equivalent dose >15 mg/day [3]. These rules, issued by the Medical Licensing Board, require prescribers to: (1) evaluate opioid recipients for psychiatric conditions; (2) review patients’ drug prescription history in INSPECT and (3) perform regular drug screenings; and (4) obtain a signed controlled-substance agreement from the patient [3]. Prescribers were to query at initiation and at least yearly.

### Time segments and participants

Data for all opioids dispensed in the state of Indiana between January 1, 2011 (1079 days before to the emergency rules) and November 6, 2014 (325 days after the policy) were obtained. Prescription level INSPECT data was merged with census data to characterize socioeconomic status for patients.

### Statistical analysis

An interrupted time series analysis (ITSA) was utilized to investigate the association between the Indiana emergency rules and opioid prescribing, controlling for time trends and the auto-regressive nature of the time series. The aggregated ITSA results in Tables 1 and 2 are for the full group of providers or patients within the state of Indiana on a given day. Individuals within this group may vary each day. ITSA relies on OLS regression models that are more flexible than ARIMA models but at the same time broadly applicable to interrupted time series context. Thus, the difference in daily prescribing between the pre- and post-intervention period can be interpreted as plausibly associated to the rule change. The primary outcome indicator is the total MED of dispensed opioids per day in the state of Indiana. Other outcomes considered include the number of unique patients, the number of unique providers, the number of prescriptions, the MED per transaction, the MED per day, and the number of days supplied. To further check that what we are capturing is a plausible impact of the policy, we tested for interruptions by replacing the true policy start date with other pseudo-start dates along the pre-intervention continuum.^1^ In addition, to control for time invariant patient and provider characteristics, we re-estimated our model using patient and provider fixed-effects. The patient and provider fixed effects analysis (Table 3) longitudinally follows a group of providers and patients before and after the policy change and the results can be interpreted as the change in opioid prescribing correlated with the change in policy.

**Table 1:** Association of Indiana’s opioid prescription emergency rules to daily Morphine Equivalent Dose (MED) per patient of opioids dispensed by patient gender, age, payer type, number of scripts within MED per day per patient brackets and by number of days of supply per prescription

	Impact of policy	Pre-policy Time Trend	Post-policy time trend
Gender			
Females	−2.80***	−.0027***	−.0042***
Males	−3.68***	−.0014***	−0.0044**
Age			
0–20 years	- 27.26***	−.0151***	0.0219***
20–40 years	−3.00***	.0009***	−0.0045***
40–60 years	−2.45***	−.0017***	−0.0023
60+ years	−2.04***	−.0014***	−0.0016
Payer Type			
Commercial Ins.	−3.32***	−.0041***	0.0001*
Medicaid	−3.96***	−.0018***	−0.0100***
Medicare	−4.03***	.0037***	−0.0080***
Private Pay	−2.65***	.0007**	−0.0019
Worker’s Comp	−5.93***	.0038***	0.0120*
# scripts within clinically relevant MED per day brackets			
0–20	41.72	.4939***	0.1299
20–40	−836.76 ***	−.1699	−0.6885
40–60	−363.90 ***	.2325*	−0.1411
60–80	−126.24***	.0072	−0.2214
80–100	−110.34***	.0511*	0.0058
100+	−345.37***	.1486*	−0.2608
# scripts within clinically relevant # days of supply brackets			
0–15	−635.96*	−.3354	−1.2997
16–30	−1077.80***	1.1207***	0.2100
> 30	−49.59***	−.0076	−0.0843**

Coefficients and t-statistics from Interrupted Time Series Analysis. *p ≤ 0.10, **p ≤ 0.05, ***p ≤ 0.01

**Table 2:** The association of Indiana’s opioid prescription emergency rules to daily Morphine Equivalent Dose (MED) per patient of different opioids dispensed in Indiana

Drug Name	Impact of policy	Pre-policy Time Trend	Post-policy time trend
All opioids	−3.17***	−0.0021***	−0.0044***
hydrocodone	−3.68***	−0.0038***	−0.0048***
oxycodone	−2.03***	−0.0015***	−0.0133***
morphine	−0.03	−.0101***	−0.0185***
methadone	−6.19***	−.0026***	−0.0333***
fentanyl	−1.15	−.0080***	−0.0169***
oxymorphone	−3.11*	−.0245***	−0.0142*
buprenorphine	−.47	−.0022***	−0.0209***
hydromorphone	−3.54**	.0005	−0.0143*

Coefficients and t-statistics from Interrupted Time Series Analysis. *p ≤ 0.10, **p ≤ 0.05, ***p ≤ 0.01

**Table 3:** Association of Indiana’s opioid prescription emergency rules to daily Morphine Equivalent Dose (MED) per patient of opioids dispensed, controlling for *patient and provider fixed effects* (*n* = 1404 days)

	Recipient fixed effects	Provider fixed effects
Impact of policy, instantaneous	−72.7***	−67.2***
Impact of policy on trend	−0.045***	−0.024

(1)*p ≤ 0.10, **p ≤ 0.05, ***p ≤ 0.01

(2) Includes controls for pre- and post-intervention time trends, patient age and age squares, gender and payer type

Next, analyses were stratified by patient gender, age groups, ranges of opioid dosages, and payers. We examined the significance of aggregate recipients’ and practices’ annual per-capita income by zip code level. We also investigated the policy’s plausible impact, within each decile of daily average MED of dispensed opioids per recipient. Finally, because specific drugs are associated with different extents of non-medical use and different patterns of prescribing, in addition to studying the impact of the rules on all opioids, we individually assessed the eight most commonly prescribed opioids: hydrocodone, oxycodone, morphine, methadone, fentanyl, oxymorphone, buprenorphine, and hydromorphone.

## Results

### Aggregate prescriptions and fixed effects

#### Aggregate prescriptions

**Fig. 1:**
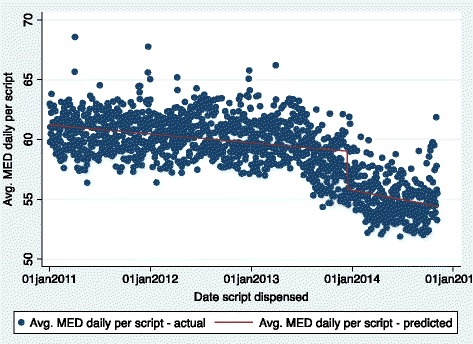
Association of Indiana opioid prescribing emergency rules with average Morphine Equivalent Dose (MED) of all opioids per day per patient. (n = 1404 days)

After controlling for time trend over our observation window, the policy is found to be statistically significantly associated with a negative instantaneous shift in MED per day and the total MED dispensed in the state (Fig. 1). We found no statistically significant ‘pseudo-policy’ effect even after evaluating a wide array of start dates. This robustness check suggests that we may be capturing a plausible impact of the emergency rules and not just an interruption in the time trend. Furthermore, we identified a second-order possible impact of the rules, captured by an additional significant negative change in the time trend in dispensed opioids. Therefore, not only were the emergency rules associated with an instantaneous downward shift in levels of opioid drugs dispensed in the short run, but also with a significant negative change in the longer term trend. The latter has a significant cumulative effect over time on curtailing the dispensing of opioids. To account for the rescheduling of tramadol in August 2014, a robustness check re-estimated the interrupted time series regression using data up-to August 17, 2014. All results were robust in both sign as well as magnitude.

#### Decile analysis

The emergency rules are not associated with a significant decline in MED (in millions) per day of dispensed opioids in the lower three deciles, but it had a statistically significant, and almost constant, negative association in the top seven deciles. This suggests that the rules plausibly did not impact very low dosages of opioid prescriptions. This is a clinically relevant finding since opioids in lower dosages are prescribed often for acute and self-limiting pain conditions.

#### Patient and prescriber fixed effects

Table 3 presents the coefficients from the fixed effects regression of daily MED per patient on implementation of the Indiana emergency rules at the patient and provider levels. This sensitivity analysis suggests that our interrupted time series results are robust to controls for patient and provider time invariant fixed characteristics, for instance physician specialization, further suggesting a plausibly causal interpretation.

### Patients and providers analysis

Table 1 provides coefficient estimates from the interrupted time series regression. Results are presented separately for daily MED per patient for different subpopulations stratified by patient gender, age, payer subtypes and number of scripts written in clinically relevant categories of number of MED (in millions) per day and by number of days of supply per prescription. A coefficient of −2.80 for females is interpreted as an instantaneous downward shift, in levels, of 2.8 MED of dispensed opioids per female patient associated with the emergency rules coming into effect.

#### Patient gender

The emergency rules are associated with a significant decline, in levels and trends, in daily MED per patient of opioids dispensed for both genders; however, the effect was quantitatively larger for men. This is interesting since women comprised a larger proportion of the patient population receiving opioids.

#### Patient age

We observed a significant decline in the daily MED per patient prescription of opioid drugs for all age groups. However, large differences in magnitude exist, with the decline being almost 10 times larger in the 0–20 age range as compared to the 60+ years age range.

#### Insurance types

Considering the differential impact of the emergency rules by payer type, overall, we found that the rules are significantly associated to a downward shift in levels of dispensed opioids, with the largest impact on drugs paid through Worker’s Compensation, followed by Medicare and Medicaid. Considering long-term changes, all payer subgroups experienced a significant decline in the trend component as well, opioids dispensed on commercial insurance being the only exception. Despite a significant instantaneous decline in dispensed opioids paid through commercial insurance, the long-term trend for this subgroup actually significantly increased in the post emergency rules period. Finally, since only a small number of prescriptions were paid for with military insurance and Veterans’ Affairs in the pre-intervention period and none in the post-intervention period, we are unable to conduct analysis for that payer sub-group.

#### Socioeconomic status of patients and providers

As expected, there is a significant negative impact of patient socioeconomic status (SES) on opioid prescribing, with higher amounts of opioid prescriptions being associated with patients with lower SES, even after controlling for the aggregate recipient annual per-capita income (Table 4). In addition, we found a significant positive correlation between aggregate provider annual per-capita income. Higher levels of opioid prescriptions were associated with higher aggregate income at the provider level, which did not change after the emergency rules.

**Table 4:** Association of Indiana’s opioid prescription emergency rules to daily Morphine Equivalent Dose (MED) per patient of opioids dispensed, controlling for aggregate socioeconomic characteristics of *patients and providers by zip code* (*n* = 1404 days)

	(1)	(2)	(3)
Impact of policy	−57.2**	7.15	−27.2
Agg. Recipient ann. Per cap. Income	−18.5***		−21.2***
Agg. Recipient ann. Per cap. Income *policy	6.86**		6.72**
Agg. provider ann. Per cap. Income		9.34***	12.8***
Agg. provider ann. Per cap. Income *policy		−1.00	−2.82

(1)*p ≤ 0.10, **p ≤ 0.05, ***p ≤ 0.01

(2) Includes controls for pre- and post-intervention time trends. (1), (2), (3), refer to the three regression models used in the analysis

#### Number of prescribers

Table 5 illustrates that fewer providers prescribed opioids after the rule change. For providers still prescribing opioid analgesics, each was writing fewer opioid prescriptions for shorter durations and for lower supply doses, on average. Since the emergency rules did not significantly predict a linear measure of MED per day, we examined the presence of any non-linear associations by regressing clinically relevant MED per-day categories on the emergency rule change dummy. The results presented in Table 2 show a striking non-linear association of the emergency rules with different MED per-day brackets. While the rules are not significantly associated to changes in prescriptions with relatively low doses of 0–20 MED per day, it predicts a significant decline in higher MED prescriptions, with particularly high declines within the 20–40 and 100+ MED per-day categories.

**Table 5:** Association of Indiana’s opioid prescription emergency rules to daily Morphine Equivalent Dose (MED) per patient of opioids dispensed, controlling for potential mediators (*n* = 1404 days)

Daily averages	Impact of policy	Coef. On potential mediator in post policy period
# providers	3.22	−.0010***
# prescriptions per provider	−.28	−.2189**
# days of supply	13.58	−.8037*
MED per day	.24	−.0109

(1)*p ≤ 0.10, **p ≤ 0.05, ***p ≤ 0.01

(2) Includes controls for pre- and post-intervention time trends

### Other outcomes

#### Day supply

We examined whether the coming into effect of the emergency rules significantly predicted the number of scripts written within different clinically relevant number of days prescribed brackets. The results are presented in Table 2. While we note a significant decline in the number of scripts written across all days of supply categories, the association of the emergency rules is once again non-linear and, in fact, U-shaped. The rules seem to predict the largest reduction in the number of scripts written with 16–30-days of supply category, with a much smaller but still significant decline in the number of longer-running prescriptions.

### Individual opioids

Regression coefficients for all opioid drugs prescribed, altogether and by specific drug types, are presented in Table 3. The emergency rules are associated with significant declines in the daily MED per patient of all dispensed opioids and also with most individual drugs. The only exceptions were morphine, fentanyl, and buprenorphine, all of which did not present a statistically significant decline post emergency rules. We investigated this further by comparing the short- and long-acting forms of morphine to the short- and long-acting forms of oxycodone, a drug that experienced a significant decline in prescription rates in the post emergency rules period. Table 6 shows that the rules predict a significant decline in the short-acting but not the long-acting form of oxycodone, and in the long-acting but not the short-acting form of morphine.

**Table 6:** Coefficients and t-statistics from Interrupted Time Series Analysis of changes in daily Morphine Equivalent Dose (MED) per patient of opioids dispensed associated with opioid emergency rules, of long and short acting oxycodone and morphine dispensed in Indiana (n = 1404 days)

Drug Name	Impact of policy	Pre-policy Time Trend	Post-policy time trend
Oxycodone, all	−2.03***	−0.0015***	−0.0133***
short	−.88***	.0023***	0.0036***
long	−.08	−.0002***	−0.0002
Morphine, all	−0.03	−.0101***	−0.0185***
short	−.08	.0004***	−0.0001
long	−.20***	.0012***	−0.0005***

(1)*p ≤ 0.10, **p ≤ 0.05, ***p ≤ 0.01)

## Discussion

Prescription drug non-medical use is a major public health concern. State mandated opioid prescribing emergency rules to change prescribing practices have been adopted across the United States; however, the impact of these rules on provider prescribing behavior has not been effectively studied. In this paper, we report that Indiana’s opioid emergency rules are associated to a decrease in the volume of total opioid prescriptions and in the average number of opioid MED per patient. It follows that with a reduction in inappropriate opioid dispensing lower rates of opioid misuse and non-medical use should also be realized. From the standpoint of both the population’s health and the individual patient’s care, these findings are of significant importance.

The results show that the emergency rules were associated with significant changes in prescribing behavior statewide and across all individual patient and provider characteristics, albeit with varying magnitudes. In particular, reduced opioid prescriptions were noted across genders, health care/insurance types, and age groups. In addition, reduced prescribing was not limited to certain types of prescriptions (high doses, longer durations, etc.). Regarding types of medications, prescriptions of nearly all opioids commonly prescribed for chronic pain declined. Prescribing rates were noticeably lower in all deciles, with the exception of the lower 30% of prescribing rates.

Prior studies have assessed if patient characteristics, such as gender, age, insurance type, and socioeconomic status are significant predictors of quality of pain management, opioid prescribing, and drug non-medical use [7, 8]. Our results show that the Indiana rules are indeed differently associated to different patient subgroups. Post emergency rules, the decline in opioid prescribing was more prominent for males compared to females, patients who have Medicare and Medicaid compared to those with private insurances, and younger patients compared to older ones. Furthermore, practicing in areas with lower incomes was associated with a greater decrease in prescribing after the policy. While it is not surprising that the new emergency rules predict steepest declines among the highest opioid recipients, other patient and provider characteristics also seem to be associated with different prescribing patterns in terms of quantities of opioids.

Fewer providers prescribed opioids after implementation of the rules, and for those still prescribing opioid analgesics, each provider wrote fewer prescriptions for shorter durations and lower doses, on average. The emergency rules are not significantly associated with changes in prescriptions with relatively low doses of 0–20 MED per day; however, they did plausibly significantly lower higher MED prescriptions. Furthermore, the largest MED reduction was observed within the 16–30 day category, with a much smaller but still significant decline in the longer-running prescriptions. Isolating such effects confirms our hypothesis that the changes in prescribing patters are possibly the result of the rules and not merely random time trends.

The emergency rules are not associated to changes in all types of opioids equally. Buprenorphine, which is used in the treatment of pain and addiction, has gained an increased popularity over the past few years. Buprenorphine, unlike methadone, is registered in INSPECT when used as buprenorphine/naloxone (Suboxone or Subutex) for addiction or as buprenorphine alone for pain management [13]. Fentanyl was another agent that did not see a reduction in use following the new opioid prescribing emergency rules. Of note, the rules did have several patient exclusions, including that they did not apply to patients with terminal medical conditions or those in Indiana-licensed hospice or palliative care programs. Although fentanyl certainly can be subject to misuse and non-medical use, its most popular formulation in an outpatient setting, the transdermal patch, is indicated for chronic pain management in opioid-tolerant patients who have had insufficient pain relief with other treatment options [14]. However, the continued use of fentanyl at rates similar to those before the emergency rules is a relevant observation in light of the rising concern of increased mortality due to fentanyl non-medical use [13]. Existing literature finds that non-medical fentanyl use and resulting fatalities are largely driven by synthetic fentanyl illegally manufactured [15]. Finally, the emergency rules predicted different magnitudes of change in the short-acting and the long-acting forms of morphine and oxycodone. Although decreases in short-acting formulations are expected post implementation of the rules, perhaps short-acting morphine remains a preferred agent in the acute pain setting (thus no reduction seen).

Our study is not without limitations. The use of time series analysis provides some evidence for causality. Stronger evidence can be identified in the presence of a comparison state. A neighboring state, such as Ohio or Kentucky, appears to be an appealing option in light of similar patient and provider demographics [16]. In future work, we hope to gain access to PDMP data from potential comparison states and conduct comparative analyses to better infer the plausibly causal impact of the emergency rules using as a control group the state(s) that appears to be the closest to Indiana in its summary statistics, but that did not implement the emergency rules yet. While our study brings attention to several socio-demographic-economic determinants of practice, the absence of important other factors, such as race and individual patient SES indicators, have limited our ability to draw strong conclusions. Further, prescriber zip codes in our data could capture location of the prescriber’s residence or practice, which limits our ability to precisely interpret the positive association between zip code aggregate socioeconomic status and opioid prescribing changes. A qualitative evaluation of a representative sample of patients and prescribers could provide further evidence in examining these factors. Such analyses would also bring to light any unintended consequences that the implementation of the emergency rules may have. For instance, the added regulatory burden of opioid prescribing following the emergency rules may deter providers from prescribing altogether, resulting in worsening of patient pain outcomes. The impact of the emergency rules on access-to-appropriate treatment and care involving opioid alternatives could not be evaluated using our current data and is left to future work. Finally, while the daily opioid dose has been linked to patient outcomes such as overdose and death, our study did not explore those outcomes. Other important outcomes include drug diversion and crime, both of which are directly linked to opioid prescribing; they can also be explored in further studies.

## Conclusion

Indiana’s opioid emergency rules are associated to a decrease in the volume of opioid prescriptions and in the average number of opioid analgesic prescriptions for individual patients. The new emergency rules, however, predict different changes for different groups. The decline in opioid prescribing was more prominent for males compared to females, patients who have Medicare and Medicaid compared to those with private insurances, and younger patients compared to older ones.

## Abbreviations

INSPECT: Indiana State Prescription Electronic Collection and Tracking
ITSA: Interrupted time series analysis
MED: Morphine equivalent dose
NDC: National drug code
PDMP: Prescription drug monitoring program
SES: Socioeconomic status

## Footnotes

1 A random number generator was used to randomly draw 500 pseudo start dates along the pre-intervention continuum.
